# Cost-Effectiveness of Annual Prostate MRI and Potential MRI-Guided Biopsy After Prostate-Specific Antigen Test Results

**DOI:** 10.1001/jamanetworkopen.2023.44856

**Published:** 2023-11-29

**Authors:** Hyunkyung Yun, Jin Kim, Aishwarya Gandhe, Brianna Nelson, Jim C. Hu, Vikas Gulani, Daniel Margolis, Bruce R. Schackman, Ali Jalali

**Affiliations:** 1 Department of Population Health Sciences, Weill Cornell Medicine, Cornell University, New York, New York; 2 Department of Health Services, Policy & Practice, School of Public Health, Brown University, Providence, Rhode Island; 3 Department of Urology, Weill Cornell Medicine, Cornell University, New York, New York; 4 Department of Radiology, University of Michigan Health System, Ann Arbor; 5 Department of Radiology, Weill Cornell Medicine, Cornell University, New York, New York

## Abstract

**Importance:**

Magnetic resonance imaging (MRI) and potential MRI-guided biopsy enable enhanced identification of clinically significant prostate cancer. Despite proven efficacy, MRI and potential MRI-guided biopsy remain costly, and there is limited evidence regarding the cost-effectiveness of this approach in general and for different prostate-specific antigen (PSA) strata.

**Objective:**

To examine the cost-effectiveness of integrating annual MRI and potential MRI-guided biopsy as part of clinical decision-making for men after being screened for prostate cancer compared with standard biopsy.

**Design, Setting, and Participants:**

Using a decision analytic Markov cohort model, an economic evaluation was conducted projecting outcomes over 10 years for a hypothetical cohort of 65-year-old men in the US with 4 different PSA strata (<2.5 ng/mL, 2.5-4.0 ng/mL, 4.1-10.0 ng/mL, >10 ng/mL) identified by screening through Monte Carlo microsimulation with 10 000 trials. Model inputs for probabilities, costs in 2020 US dollars, and quality-adjusted life-years (QALYs) were from the literature and expert consultation. The model was specifically designed to reflect the US health care system, adopting a federal payer perspective (ie, Medicare).

**Exposures:**

Magnetic resonance imaging with potential MRI-guided biopsy and standard biopsy.

**Main Outcomes and Measures:**

Incremental cost-effectiveness ratios (ICERs) using a willingness-to-pay threshold of $100 000 per QALY was estimated. One-way and probabilistic sensitivity analyses were performed.

**Results:**

For the 3 PSA strata of 2.5 ng/mL or greater, the MRI and potential MRI-guided biopsy strategy was cost-effective compared with standard biopsy (PSA 2.5-4.0 ng/mL: base-case ICER, $21 131/QALY; PSA 4.1-10.0 ng/mL: base-case ICER, $12 336/QALY; PSA >10.0 ng/mL: base-case ICER, $6000/QALY). Results varied depending on the diagnostic accuracy of MRI and potential MRI-guided biopsy. Results of probabilistic sensitivity analyses showed that the MRI and potential MRI-guided biopsy strategy was cost-effective at the willingness-to-pay threshold of $100 000 per QALY in a range between 76% and 81% of simulations for each of the 3 PSA strata of 2.5 ng/mL or more.

**Conclusions and Relevance:**

This economic evaluation of a hypothetical cohort suggests that an annual MRI and potential MRI-guided biopsy was a cost-effective option from a US federal payer perspective compared with standard biopsy for newly eligible male Medicare beneficiaries with a serum PSA level of 2.5 ng/mL or more.

## Key Points

**Question:**

Are an annual prostate magnetic resonance imaging (MRI) and potential MRI-guided biopsy cost-effective compared with a transrectal ultrasonographic (standard) biopsy for men with a positive prostate-specific antigen (PSA) test result?

**Findings:**

In this economic evaluation, annual MRI and potential MRI-guided biopsy for prostate cancer found improved economic value compared with standard biopsy for men with a serum PSA level of 2.5 ng/mL or more beginning at 65 years of age.

**Meaning:**

This study suggests that an annual MRI and potential MRI-guided biopsy are a cost-effective option from the federal payer perspective compared with standard biopsy for men beginning at 65 years of age for a serum PSA level of 2.5 ng/mL or more.

## Teaser

This economic evaluation compares the cost-effectiveness of integrating annual magnetic resonance imaging and potential magnetic resonance imaging–guided biopsy vs standard biopsy as part of clinical decision-making for men after being screened for prostate cancer.

## Introduction

Prostate cancer (PCa) is the most common noncutaneous cancer among men in the US and the second-highest cause of cancer deaths among men.^1,2^ There is evidence that significant cost savings can be achieved by enhanced precision in diagnosis.^3,4^ Magnetic resonance imaging (MRI) and MRI-guided biopsy have been introduced for the diagnosis of PCa. The standard for detecting PCa is transrectal ultrasonography (TRUS)–guided biopsy with 12 to 16 cores distributed throughout the prostate (standard biopsy).^5^ This approach is associated with risks of postbiopsy complications,^6^ has limited diagnostic accuracy,^7^ and is performed without visual confirmation of the cancer’s location.^8^ The primary diagnostic limitations of standard biopsies are overdetection of clinically insignificant lower-grade PCa and underdetection or undergrading of clinically significant PCa.^9^ There remains substantial controversy regarding prostate-specific antigen (PSA) screening for PCa, particularly its potential to result in overdiagnosis of low-risk disease.^10^ Despite these concerns, PSA screening remains a commonly used tool, and both American Urological Association and American Cancer Society guidelines continue to include PSA screening for PCa diagnosis.^11,12,13^

Multiparametric MRI can assess which men have regions suspicious for cancer in the prostate, improving detection of clinically significant cancer and potentially avoiding biopsy when no target is evident.^14^ Despite proven efficacy, MRI and potential MRI-guided biopsy remain costly, and there is limited research evaluating the cost-effectiveness of this approach.^5,15^

We evaluate the cost-effectiveness of annual MRI and potential MRI-guided biopsy as a screening strategy for PCa detection compared with standard biopsy. We focused on newly eligible male Medicare beneficiaries who are 65 years of age over 10 years to maintain a model consistent with the initial phase of Medicare eligibility to the age limit beyond which PCa screening is generally not recommended.^16^

## Methods

### Model Overview

In this economic evaluation, a decision analytic Markov model based on current US epidemiologic and clinical data was constructed to evaluate the cost and effectiveness of 2 biopsy strategies: standard biopsy and MRI and potential MRI-guided biopsy in the US (eFigure 1 in Supplement 1). The model was constructed from a federal payer (Medicare) perspective and developed to evaluate outcomes based on prostate MRI–naive men entering the model at an age of 65 years over a 10-year time horizon, which is the earliest age that men undergoing PSA screening would be eligible for Medicare; nearly 66% of PCa cases are diagnosed among men aged 65 years or older,^17,18^ while men older than 75 years are less likely to undergo definitive therapy for PCa because they face greater morbidity and mortality risk associated with other health issues.^19^ The study was conducted using simulated data and does not constitute human participants research and is therefore exempt from institutional review board approval. We followed the Consolidated Health Economic Evaluation Reporting Standards (CHEERS) reporting guideline.

The Markov model uses annual transition probabilities to model a hypothetical individual’s simulated history as they progress between multiple health states, factoring in health utilities and costs associated with treatment complications, mortality, and other relevant events (eFigure 1 in Supplement 1). At the end of each cycle (annual), costs and quality-of-life weights incurred in each health state are summarized and aggregated for each strategy.

The population was categorized by 4 initial PSA strata: less than 2.5 ng/mL, 2.5 to 4.0 ng/mL, 4.1 to 10.0 ng/mL, and more than 10.0 ng/mL (to convert to micrograms per liter, multiply by 1.0). Without loss of generality, the models assume that an individual’s PSA category would not change over the 10-year horizon and have identical structures but with different probabilities of PCa recurrence due to disease progression. The aggressiveness of PCa is described by pathologic grade, stratified to grade groups based on the preponderance of histologic Gleason patterns.^20^

The model accounts for the sensitivity in detecting clinically significant PCa and specificity of potential MRI-guided biopsy (sensitivity, 0.900 [range, 0.644-0.997]; specificity, 1.000 [range, 0.906-1.000]) and standard biopsy (sensitivity, 0.760 [range, 0.488-0.929]; specificity, 0.960 [range, 0.740-1.000]) and the sensitivity or specificity of MRI (sensitivity, 0.760 [range, 0.528-0.911]; specificity, 0.880 [range, 0.622-0.989])^5,21,22^; positive and negative results, as well as false-positive and false-negative results, are recorded. If MRI detects a possible PCa, individuals will receive an MRI-guided biopsy. If the MRI findings are negative, no biopsy is conducted.

Patients with comorbidities such as diabetes, chronic obstructive pulmonary disease, congestive heart failure, or multiple comorbid conditions that can be exacerbated by treatment may defer PCa treatment out of concern that it would not improve length or quality of life.^23^ Those with high-grade PCa, however, may require treatment regardless of the presence of comorbidities.^24^

Descriptive and structural validity was conducted between the economic and clinical authors first and then by the modeling team prior to evaluating final economic outcomes. Using the complete model, internal validity and consistency were checked via multiple sensitivity analyses by assessing the association of varying key parameter values, such as diagnostic test characteristics and cancer progression probabilities, with variation in relative Markov cohort sizes from one cycle to another. Additional model details required to replicate this study are included in the eAppendix in Supplement 1.

### Data Sources

Data were derived from the National Vital Statistics Report of the US Centers for Disease Control and Prevention, the Medicare fee schedule, published research, and expert consultation with medical professionals (J.C.H, V.G., and D.M.). These sources provided transition probabilities,^5,21,22,25,26,27,28,29^ costs, and quality-of-life weights^3,4,27,30,31,32,33,34,35,36,37^ associated with each health state in the Markov model (Table 1^5,21,22,25,26,27,28,29^ and Table 2^3,4,27,30,31,32,33,34,35,36,37^; eTable in Supplement 1). All costs were adjusted to 2020 US dollars. Cost for in-bore MRI-targeted biopsy was used to assess economic end points, establish the upper-bound cost for biopsy options (as MRI-TRUS fusion and cognitive targeting are expected to cost less), and be conservative in our analysis of the MRI-guided biopsy strategy. Additional detail on data sources and derivation of input parameters is included in the eAppendix in Supplement 1.

**Table 1.** PCa and Test-Related Probability Inputs^a^

Parameter	Distribution	Value (range)	Reference
Probability of having PCa in PSA <2.5 ng/mL stratum	Beta	0.020 (0.001-0.082)	Gretzer and Partin,^25^ 2002
Probability of having PCa in PSA 2.5-4.0 ng/mL stratum	Beta	0.180 (0.143-0.225)	Gretzer and Partin,^25^ 2002
Probability of having PCa in PSA 4.1-10.0 ng/mL stratum	Beta	0.300 (0.260-0.340)	Gretzer and Partin,^25^ 2002; clinical judgement
Probability of having PCa in PSA >10.0 ng/mL stratum	Beta	0.670 (0.630-0.700)	Gretzer and Partin,^25^ 2002
Probability of PCa recurrence in PSA <2.5 ng/mL stratum	Beta	0.008 (0-0.072)	Xia et al,^26^ 2014
Probability of PCA recurrence in PSA 2.5-4.0 ng/mL stratum	Beta	0.014 (0.000-0.050)	Xia et al,^26^ 2014
Probability of PCa recurrence in PSA 4.1-10.0 ng/mL stratum	Beta	0.026 (0.007-0.058)	Xia et al,^26^ 2014
Probability of PCa recurrence in PSA >10.0 ng/mL stratum	Beta	0.066 (0.041-0.094)	Xia et al,^26^ 2014
Probability of having adverse effect from MRI-guided biopsy	Beta	0.400 (0.226-0.611)	Egbers et al,^27^ 2015
Probability of having adverse effect from standard biopsy	Beta	0.600 (0.413-0.786)	Egbers et al,^27^ 2015
Probability of having high-grade PCa without comorbidities	Beta	0.139 (0.020-0.395)	Hjälm-Eriksson et al,^28^ 2017
Probability of having high-grade PCa with comorbidities	Beta	0.139 (0.026-0.424)	Hjälm-Eriksson et al,^28^ 2017
Probability of having intermediate-grade PCa without comorbidities	Beta	0.308 (0.150-0.508)	Hjälm-Eriksson et al,^28^ 2017
Probability of having intermediate-grade PCa with comorbidities	Beta	0.332 (0.171-0.549)	Hjälm-Eriksson et al,^28^ 2017
Probability of having low-grade PCa without comorbidities	Beta	0.043 (0.001-0.231)	Hjälm-Eriksson et al,^28^ 2017
Probability of having low-grade PCa with comorbidities	Beta	0.039 (0.000-0.251)	Hjälm-Eriksson et al,^28^ 2017
Probability of upgrading in PCa grades	Beta	0.250 (0.094-0.460)	Chang et al,^29^ 2018; clinical judgement
Sensitivity of MRI	Beta	0.760 (0.528-0.911)	Pahwa et al,^5^ 2017
Specificity of MRI	Beta	0.880 (0.622-0.989)	Pahwa et al,^5^ 2017
Sensitivity of potential MRI-guided biopsy	Beta	0.900 (0.644-0.997)	Shoji,^21^ 2019
Specificity of potential MRI-guided biopsy	Beta	1.000 (0.906-1.000)	Clinical judgement
Sensitivity of standard biopsy	Beta	0.760 (0.488-0.929)	Shoji,^21^ 2019
Specificity of standard biopsy	Beta	0.960 (0.740-1.000)	Streicher et al,^22^ 2019

Abbreviations: MRI, magnetic resonance imaging; PCa, prostate cancer; PSA, prostate-specific antigen.

SI conversion factor: To convert PSA to micrograms per liter, multiply by 1.0.

^a^ The probability of PCa recurrence for different PSA strata is derived from Xia et al,^26^ and the probability for the PSA 4.1 to 10.0 ng/mL stratum was found using a mean probability of the PSA 4.1 to 6.0 ng/mL and PSA 6.1 to 10.0 ng/mL strata, and the values were calculated to annual probabilities. The probabilities of having adverse effects are the means of having adverse effects including pain, bleeding, hematuria, hemospermia, and rectal hemorrhage. The probabilities of having different risk grade of PCa by the presence of comorbid conditions were calculated using the values from Hjälm-Eriksson et al.^28^ The probability of upgrading in PCa averaged 26.9% for Gleason score 3 + 3 and 22.6% for Gleason score 3 + 4, and the range of the variable was derived from the ranges of the 2 Gleason score levels as well. Values in the range column were collected from the probabilistic sensitivity analyses results.

**Table 2.** Cost and Health Utility Weight Inputs^a^

Intervention	Distribution	Value (range)	Reference
PSA screening	Gamma	$18 ($7 to $38)	Medicare data
Standard biopsy	Gamma	$474 ($173 to $913)	Medicare data
MRI	Gamma	$560 ($212 to $1089)	Medicare data
Potential MRI-guided biopsy	Gamma	$723 ($315 to $1445)	Medicare data
Treatment			
Prostatectomy	Gamma	$16 306 ($5881 to $31 282)	Medicare data; Aizer et al,^3^ 2015
Radiotherapy	Gamma	$21 860 ($8841 to $45 305)	Medicare data; Aizer et al,^3^ 2015
Brachytherapy	Gamma	$19 792 ($7981 to $38 009)	Medicare data; Aizer et al,^3^ 2015
Treatment management			
Health care costs for patients with comorbidities and untreated PCa^b^	Gamma	$706 ($284 to $1343)	Medicare data; Trogdon et al,^4^ 2019
Health care costs after treatment^c^	Gamma	$1353 ($531 to $2560)	Medicare data; Cooperberg et al,^30^ 2013
Health state			
Healthy without PCa	NA	1.000 (no range)	Roth et al,^31^ 2016
High-grade PCa	Beta	−0.200 (−0.430 to −0.047)	Heijnsdijk et al,^32^ 2012
Low- or intermediate-grade PCa	Beta	−0.030 (−0.079 to −0.005)	Heijnsdijk et al,^32^ 2012
PCa plus comorbidity	Beta	−0.233 (−0.440 to −0.066)	Sullivan et al,^33^ 2005
PCa recurrence	Beta	−0.320 (−0.550 to −0.150)	Lobo et al,^34^ 2017
PSA screening	Beta	−0.0002 (−0.001 to −0.000)	Barnett et al,^35^ 2018
Adverse effect of standard biopsy	Beta	−0.016 (−0.090 to −0.000)	Barnett et al,^35^ 2018
Adverse effect of potential MRI-guided biopsy	Beta	−0.016 (−0.090 to −0.000)	Egbers et al,^27^ 2015
Prostatectomy	Beta	−0.250 (−0.460 to −0.085)	de Carvalho et al,^36^ 2018
Radiotherapy	Beta	−0.230 (−0.450 to −0.073)	de Carvalho et al,^36^ 2018
Brachytherapy	Beta	−0.040 (−0.470 to −0.000)	Hayes et al,^37^ 2013
Quality of life after treatment by year	Beta	Year 1 (transient effectiveness), −0.119 (−0.420 to −0.009); year 2 (after the year of the treatment), −0.050 (−0.092 to −0.020)	de Carvalho et al,^36^ 2018

Abbreviations: MRI, magnetic resonance imaging; NA, not applicable; PCa, prostate cancer; PSA, prostate-specific antigen.

^a^ Pain scores of MRI-guided biopsy and standard biopsy from Egbers et al^27^ and the disutility of standard biopsy from Barnett et al^35^ were used to calculate the estimated disutility of MRI-guided biopsy by dividing the disutility of standard biopsy by the weighted mean of the pain scores ratio of the 2 biopsies. The disutility caused in the year of having treatment was calculated by averaging the disutilities of having a radical prostatectomy and radiotherapy, and the postrecovery value was used for the disutility after the year of the treatment. Values in the range column are based on distributions from the probabilistic sensitivity analyses results. The following Healthcare Common Procedure Coding System codes were used for prostate cancer screening costs: (1) cost of MRI: 72197, 76498; (2) cost of MRI-guided biopsy: 55700, 77021; (3) cost of PSA screening: 84153; and (4) cost of standard biopsy or transrectal ultrasonography: 55700, 76872, and 76942.

^b^ Annual health care cost was based on a median of 3 years of total costs incurred for patients with prostate cancer and comorbidities.

^c^ Health care cost after treatment is an annual cost.

### Statistical Analysis

#### Primary Measures

The incremental cost-effectiveness ratio (ICER) was used to assess cost-effectiveness. ICERs were calculated as the difference in costs divided by the difference in quality-adjusted life-years (QALYs) between the 2 competing strategies. All future costs and QALYs were discounted at a rate of 3% annually.^38^ We identified the efficient strategy for each PSA group using a standard willingness-to-pay (WTP) threshold of $100 000 per QALY; if the ICER was under $100 000 per QALY and the incremental QALY was positive, the strategy was considered cost-effective.^39^

#### Sensitivity Analyses

A probabilistic sensitivity analysis was performed using Monte Carlo microsimulation with 10 000 trials to account for simultaneous uncertainty in model parameter values when calculating ICERs, and 1-way sensitivity analyses of key test characteristics (sensitivity and specificity of MRI and MRI-guided biopsy) were evaluated within these simulations. In addition, we generated tornado diagrams to describe the association of uncertainty of input parameters with incremental effectiveness and cost outcomes between strategies. All analyses were performed using TreeAge Pro, version 2021 R.12 (TreeAge Software LLC). Additional detail on primary measures is included in the eAppendix in Supplement 1.

## Results

### Base-Case Scenario

The costs, QALYs, incremental values, and ICERs comparing the 2 strategies—the MRI and MRI-guided biopsy vs the standard biopsy—for the 4 different PSA strata are summarized in Table 3. In the base-case scenario, the MRI and potential MRI-guided biopsy strategy was a cost-effective strategy compared with the standard biopsy for 3 of the 4 categories of PSA strata. The ICER was well below the WTP threshold of $100 000 per QALY for these 3 PSA strata, at $21 131 per QALY for a PSA of 2.5 to 4.0 ng/mL, $12 336 per QALY for a PSA of 4.1-10.0 ng/mL, and $6000 per QALY for a PSA of more than 10.0 ng/mL. The ICER for a PSA less than 2.5 ng/mL was $187 558 per QALY. We found that the MRI and potential MRI-guided biopsy strategy improved in economic and clinical value as PSA increased. Although the total mean costs increased for both strategies for higher PSA strata, the incremental costs for the MRI and potential MRI-guided biopsy strategy decreased. Similarly, the incremental QALYs were found to increase for the MRI and potential MRI-guided biopsy strategy compared with the standard biopsy as the PSA strata increased.

**Table 3.** Cost-Effectiveness Results

PSA stratum	Expected costs, $	Incremental costs, $^a^	Expected QALYs	Incremental QALYs^a^	ICER, $/QALY^a^
**<2.5 ng/mL**					
Standard biopsy strategy	5759	NA	7.899	NA	NA
MRI and potential MRI-guided biopsy strategy	7023	1264	7.906	0.007	187 558
**2.5-4.0 ng/mL**					
Standard biopsy strategy	13 590	NA	7.373	NA	NA
MRI and potential MRI-guided biopsy strategy	14 316	726	7.407	0.034	21 131
**4.1-10.0 ng/mL**					
Standard biopsy strategy	15 429	NA	7.039	NA	NA
MRI and potential MRI-guided biopsy strategy	15 985	556	7.084	0.045	12 336
**>10.0 ng/mL**					
Standard biopsy strategy	16 872	NA	6.509	NA	NA
MRI and potential MRI-guided biopsy strategy	17 246	374	6.572	0.062	6000

Abbreviations: ICER, incremental cost-effectiveness ratio; MRI, magnetic resonance imaging; NA, not applicable; PSA, prostate-specific antigen; QALY, quality-adjusted life-year.

SI conversion factor: To convert PSA to micrograms per liter, multiply by 1.0.

^a^ Incremental values and ICERs are in reference to standard strategy.

### Sensitivity Analyses

We conducted 1-way sensitivity analyses on the test characteristics of the MRI and potential MRI-guided biopsy. Holding the sensitivity of MRI-guided biopsy constant, QALYs for the MRI and potential MRI-guided biopsy strategy decreased at very high sensitivity values of MRI. No additional incremental QALYs were gained for the MRI and potential MRI-guided biopsy strategy, with sensitivity values for the MRI greater than 0.84 across all PSA strata compared with 0.76 in the base case.

There were approximately 0.062 QALYs gained on average throughout the entire range of specificity values of MRI for the PSA stratum greater than 10 ng/mL, while the cost of the MRI and potential MRI-guided biopsy strategy decreased as the specificity of the MRI increased, resulting in an ICER less than $11 000 per QALY for the entire range. For the PSA stratum of 4.1 to 10.0 ng/mL, the ICER stayed below $50 000 per QALY for the entire range as the specificity increased. For the PSA stratum of 2.5 to 4.0 ng/mL, the specificity of the MRI needed to be at least 0.50 for the ICER to be less than $50 000 per QALY.

Holding the specificity of the MRI constant, the ICER remained at approximately $6000 per QALY or less for the entire range of specificity values of MRI-guided biopsy for the PSA stratum of more than 10 ng/mL. For the PSA stratum of 4.1 to 10.0 ng/mL and the PSA stratum of 2.5 to 4.0 ng/mL, the ICER stayed below $13 000 per QALY and $22 000 per QALY, respectively. For the PSA stratum of less than 2.5 ng/mL, the ICER decreased as the specificity increased, but it remained between $187 000 and $192 000 per QALY. Additional sensitivity analyses (eg, tornado diagrams) are provided in eFigure 2A and B in Supplement 1.

Results from probabilistic sensitivity analysis indicate that the MRI and potential MRI-guided biopsy strategy was cost-effective at the $100 000-per-QALY threshold in a range between 76% and 81% of simulations for each of the 3 PSA strata of 2.5 ng/mL or more (Figure). Throughout the range of cost-effectiveness thresholds, the probability that the MRI and potential MRI-guided biopsy was cost-effective compared with the standard biopsy was found to be greater for higher PSA strata.

**Figure.**
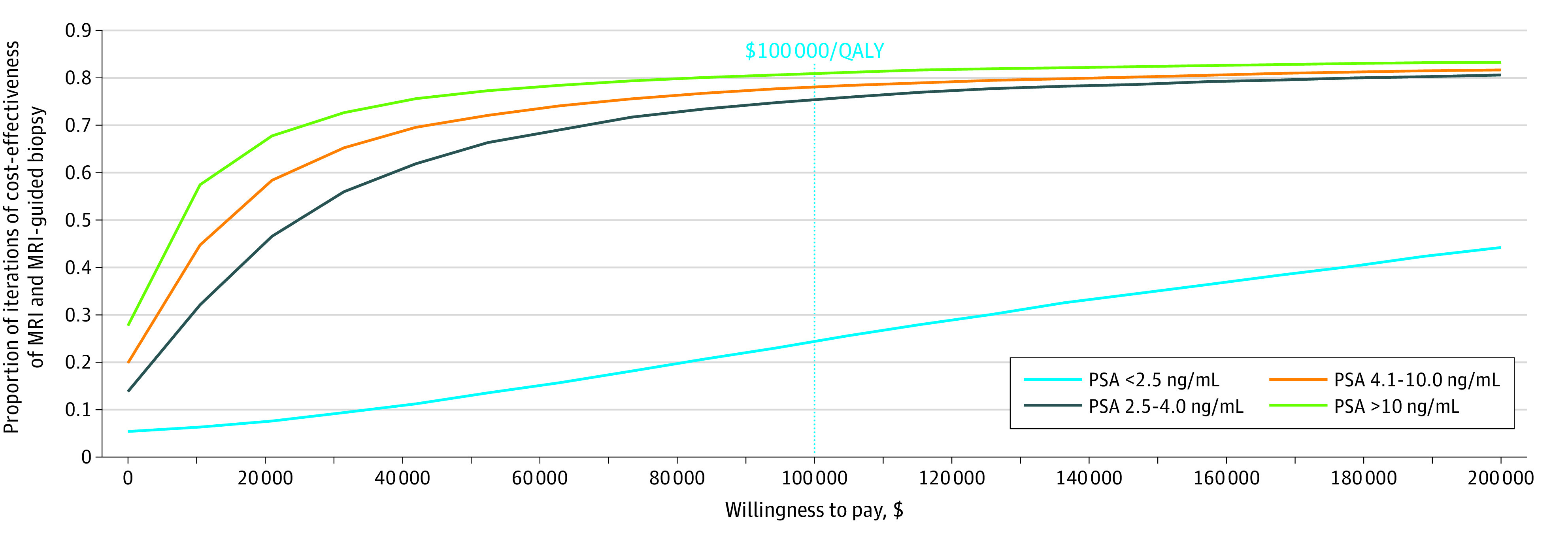
Cost-Effectiveness Acceptability Curves for Each Prostate-Specific Antigen (PSA) Stratum MRI indicates magnetic resonance imaging; and QALY, quality-adjusted life-year. To convert PSA to micrograms per liter, multiply by 1.0.

## Discussion

This economic evaluation examined the cost-effectiveness of integrating annual MRI and potential MRI-guided biopsy compared with standard biopsy differentiated by PSA strata after PCa screening in the Medicare-eligible cohort. Results from this study suggest that the MRI and potential MRI-guided biopsy strategy is cost-effective compared with standard biopsy for the detection of PCa from a federal payer perspective among 65-year-old men with a PSA level of 2.5 ng/mL or more at generally accepted WTP levels. Although for these men the initial cost of the MRI and potential MRI-guided biopsy strategy was higher than the cost of standard biopsy, extra costs were offset by higher expected QALYs. Our results are consistent with previous literature on the cost-effectiveness of MRI and potential MRI-guided biopsy strategy as management tools for PCa,^35^ in addition to its already proven value in decreasing cancer overdiagnosis demonstrated in 2 prospective randomized clinical trials.^7,40^ Findings from our study are supported by 2 prior economic evaluations that compared MRI-guided biopsy with alternative strategies, including multiparametric MRI, which also found that MRI-guided biopsy is more cost-effective than the standard of care using TRUS biopsy.^41,42^ However, PSA levels were not differentiated in those analyses. Barnett et al^35^ examined the cost-effectiveness of PCa screening strategies for people with PSA levels above 4 ng/mL and found that MRI and a combined biopsy in which both a standard biopsy and targeted MRI and ultrasonography fusion biopsy are performed is the optimal strategy, with an ICER of $23 483 per QALY. Pahwa et al^5^ found that a standard biopsy is dominated by the MRI and cognitive-guided biopsy with an ICER of –$8946 per QALY and by the MRI and in-gantry MRI-guided biopsy with an ICER of –$1263 per QALY. Our study stratifies the economic evaluation model by PSA, allowing us to examine MRI and potential MRI-guided biopsy outcomes in the context of this common marker, and we report ICERs using QALYs whose interpretation is broadly applicable. However, 1-way sensitivity analyses demonstrated no incremental QALY gain for MRI and potential MRI-guided biopsy for MRI sensitivity values greater than 0.84. This finding can be explained by the potential greater quality-of-life losses associated with early treatment compared with the risks and benefits associated with later treatment. We also account for both comorbidities and different grades of cancer in combination with PSA ranges. To our knowledge, our study is also the first economic evaluation that considered an annual MRI surveillance strategy. Prior studies have assessed the cost-effectiveness of MRI for PCa based on a single instance of screening, not accounting for costs of repeat MRI for patients receiving surveillance that occurs in clinical practice. Also, with increasing surveillance being associated with more frequent detections at lower PSA levels, especially in the 2.5 to 4.0 ng/mL range, there is a concern about the cost-effectiveness of MRI due to the lower likelihood of cancer. However, even for men in this PSA stratum, our results indicated economic value gains for stakeholders.

A concern could be raised that cancers detected by MRI strategies could be more indolent than those detected by TRUS biopsy alone, and thus stratification data from TRUS biopsy could overestimate survival benefit from an MRI-guided strategy. However, the PROMIS (Prostate Magnetic Resonance Imaging Study) trial has previously shown that cancers missed by MRI strategies are smaller and lower in grade than those detected and, thus, the opposite is expected to be true.^41^

### Limitations

As is the case in all simulation studies, this study has a number of limitations. First, in estimating treatment cost, we applied equal proportions of the 3 most common treatment options for PCa. Although changes in the proportion of treatment options will be associated with absolute cost estimates, varying the proportion of treatment will not be associated with the comparative results of the economic evaluation because any variation in cost or effectiveness (ie, treatment-related utility and survival) due to treatment proportions would be associated with both strategies equally. Furthermore, treatment may vary and change over time (and by region) depending on clinician and patient preferences,^43^ and incorporating such trends in economic models is important for research focused on budgetary effects. Future cost-effectiveness analyses of MRI and potential MRI-guided biopsy to screen for PCa should consider emerging treatment options and develop region-specific models that can more accurately project total cost and effectiveness outcomes to better inform local policy makers.

Developing a model necessitates making choices that may affect results and limit generalizability. We chose to use the cost for in-bore MRI-targeted biopsy instead of systematic TRUS fusion biopsy, as the former would detect the higher-risk grade PCa sooner, resulting in an expected increase in short-term costs and potentially decreasing the likelihood of cost-effectiveness. However, these costs may be offset by potential long-term cost reductions due to greater efficacy of treatment when PCa is detected at an early stage, which may be of particular interest to policy makers. Future studies can address the difference in the estimated economic value of these different techniques once reliable longitudinal data are available. In addition, our outcomes may vary for other reimbursement settings and perspectives. A societal perspective, for example, could result in higher time costs for the patient or other costs not covered by Medicare that may differ between the strategies.^44^ Furthermore, our study assumes that men will stay in their original PSA stratum, which may not occur naturally with aging. However, because our results provide economic and effectiveness outcomes for each PSA category, concerns regarding dynamic changes in PSA can be addressed by weighting the results of PSA categories to calculate expected values for a model that incorporated those possible variations. We expect that including changes in PSA strata over time would not result in a qualitatively different conclusion because in our analysis the MRI and potential MRI-guided biopsy strategy are optimal in all groups with a PSA level of 2.5 ng/mL or more.^45^

Our findings represent the widening of the PSA threshold from previous research by Shakir et al,^46^ which found that MRI-guided biopsy was optimal compared with standard biopsy in detecting clinically significant PCa among men with a PSA level of more than 5.2 ng/mL. In addition, the likelihood of clinically significant cancer increases with increasing serum PSA value, although this is a multifactorial phenomenon, as prostatic hyperplasia is more likely to result in an elevated PSA, and prostatic hyperplasia would decrease the sensitivity of standard biopsy. For simplicity, this scenario was not incorporated into the model.

Our study was restricted to the Medicare-eligible cohort starting at 65 years of age for men followed up for 10 years after PSA screening. This cohort is particularly relevant for policy makers not only because of their increased risk of developing PCa but also because economic modeling tends to devalue later-life outcomes (ie, discounting), and thus the evaluation of surveillance strategies for PCa will undervalue cost reductions and QALY gains that occur during the age at which the disease burden is highest. Our choice of beginning the simulation at 65 years of age minimized the impact of such time discounting by focusing on the group with the highest incidence rate. Nevertheless, further investigation may be appropriate for younger men, using a model where initial screening costs would be incurred by other payers, and the association with quality of life might extend beyond 10 years. Adjusting for the follow-up frequency, such as every other year rather than annual testing, could also be explored for individuals at low risk of PCa.

We did not further stratify our model by other patient characteristics such as family history, ethnic origin, diet, or genetic factors. This would require a markedly different model from the one used here as well as the availability of detailed longitudinal information on such cohorts. Finally, our study assumed a biopsy-naive population at enrollment. Further study is needed to determine optimal screening options for patients with a prior history of PCa or prior negative biopsy results, which would substantially alter the likelihood of receiving a diagnosis of clinically significant cancer.

## Conclusions

This economic evaluation of a hypothetical cohort from a federal payer perspective suggests that investments in expanding the capacity to conduct MRI and potential MRI-guided biopsy will provide economic value for stakeholders and improve quality of life for 65-year-old men with a PSA of 2.5 ng/mL or more undergoing screening. Results from this study add to a growing consensus that the use of MRI and potential MRI-guided biopsy is cost-effective compared with standard strategies. These findings are valuable as they can support establishing a new approach for men in active surveillance for PCa. The capacity for all men in the US to receive MRI and potential MRI-guided biopsy has been expanding, but greater adoption of this strategy is contingent on MRI availability and adequate resources and training for the MRI-guided procedures, as well as overcoming fixed cost barriers.^47^

## Notes

Supplement 1. **eAppendix.** Details on Material and Methods
eReferences.
**eTable.** Mortality Inputs by PSA Strata Used in the Decision-Analytic Model **eFigure 1.** Decision-Analytic Models **eFigure 2.** Tornado Diagrams for PSA 2.5-4.0 ng/mL Stratum

Supplement 2.
Data Sharing Statement

## References

[1] Siegel RL, Miller KD, Fuchs HE, Jemal A. Cancer statistics, 2022. CA Cancer J Clin. 2022;72(1):7-33. doi:10.3322/caac.21708 35020204

[2] American Cancer Society. Facts & Figures 2022. American Cancer Society; 2022.

[3] Aizer AA, Gu X, Chen MH, . Cost implications and complications of overtreatment of low-risk prostate cancer in the United States. J Natl Compr Canc Netw. 2015;13(1):61-68. doi:10.6004/jnccn.2015.0009 25583770

[4] Trogdon JG, Falchook AD, Basak R, Carpenter WR, Chen RC. Total Medicare costs associated with diagnosis and treatment of prostate cancer in elderly men. JAMA Oncol. 2019;5(1):60-66. doi:10.1001/jamaoncol.2018.3701 30242397 PMC6439776

[5] Pahwa S, Schiltz NK, Ponsky LE, Lu Z, Griswold MA, Gulani V. Cost-effectiveness of MR imaging–guided strategies for detection of prostate cancer in biopsy-naive men. Radiology. 2017;285(1):157-166. doi:10.1148/radiol.2017162181 28514203 PMC5621719

[6] Nam RK, Saskin R, Lee Y, . Increasing hospital admission rates for urological complications after transrectal ultrasound guided prostate biopsy. J Urol. 2010;183(3):963-968. doi:10.1016/j.juro.2009.11.043 20089283

[7] Kasivisvanathan V, Rannikko AS, Borghi M, ; PRECISION Study Group Collaborators. MRI-targeted or standard biopsy for prostate-cancer diagnosis. N Engl J Med. 2018;378(19):1767-1777. doi:10.1056/NEJMoa1801993 29552975 PMC9084630

[8] Verma S, Choyke PL, Eberhardt SC, . The current state of MR imaging–targeted biopsy techniques for detection of prostate cancer. Radiology. 2017;285(2):343-356. doi:10.1148/radiol.2017161684 29045233 PMC5673043

[9] Ahmed HU, El-Shater Bosaily A, Brown LC, ; PROMIS study group. Diagnostic accuracy of multi-parametric MRI and TRUS biopsy in prostate cancer (PROMIS): a paired validating confirmatory study. Lancet. 2017;389(10071):815-822. doi:10.1016/S0140-6736(16)32401-1 28110982

[10] Kalavacherla S, Riviere P, Javier-DesLoges J, . Low-value prostate-specific antigen screening in older males. JAMA Netw Open. 2023;6(4):e237504. doi:10.1001/jamanetworkopen.2023.7504 PMC10091155 37040113

[11] Wei JT, Barocas D, Carlsson S, . Early detection of prostate cancer: AUA/SUO guideline part I: prostate cancer screening. J Urol. 2023;210(1):46-53. doi:10.1097/JU.0000000000003491 37096582 PMC11060750

[12] Wei JT, Barocas D, Carlsson S, . Early detection of prostate cancer: AUA/SUO guideline part II: considerations for a prostate biopsy. J Urol. 2023;210(1):54-63. doi:10.1097/JU.0000000000003492 37096575 PMC11321723

[13] American Cancer Society. American Cancer Society recommendations for prostate cancer early detection. Accessed September 20, 2023. https://www.cancer.org/cancer/types/prostate-cancer/detection-diagnosis-staging/acs-recommendations.html

[14] Bjurlin MA, Meng X, Le Nobin J, . Optimization of prostate biopsy: the role of magnetic resonance imaging targeted biopsy in detection, localization and risk assessment. J Urol. 2014;192(3):648-658. doi:10.1016/j.juro.2014.03.117 24769030 PMC4224958

[15] Willis SR, van der Meulen J, Valerio M, Miners A, Ahmed HU, Emberton M. A review of economic evaluations of diagnostic strategies using imaging in men at risk of prostate cancer. Curr Opin Urol. 2015;25(6):483-489. doi:10.1097/MOU.0000000000000220 26372036

[16] Grossman DC, Curry SJ, Owens DK, ; US Preventive Services Task Force. Screening for prostate cancer: US Preventive Services Task Force recommendation statement. JAMA. 2018;319(18):1901-1913. doi:10.1001/jama.2018.3710 29801017

[17] Shah N, Ioffe V. Frequency of Gleason score 7 to 10 in 5100 elderly prostate cancer patients. *Rev Urol*. 2016;18(4):181-187. doi:10.1177/1756287219870074 28127259 PMC5260947

[18] Surveillance, Epidemiology, and End Results Program. Cancer stat facts: prostate cancer: percent of new cases by age group. National Cancer Institute. Accessed September 21, 2021. https://seer.cancer.gov/statfacts/html/prost.html

[19] National Comprehensive Cancer Network. NCCN Clinical Practice Guidelines in Oncology (NCCN Guidelines): prostate cancer early detection. January 5, 2021. Accessed February 8, 2021. https://www.nccn.org/professionals/physician_gls/pdf/prostate_detection.pdf

[20] American Cancer Society. Your Prostate pathology report: cancer (adenocarcinoma). Accessed September 20, 2021. https://www.cancer.org/treatment/understanding-your-diagnosis/tests/understanding-your-pathology-report/prostate-pathology/prostate-cancer-pathology.html

[21] Shoji S. Magnetic resonance imaging–transrectal ultrasound fusion image–guided prostate biopsy: current status of the cancer detection and the prospects of tailor-made medicine of the prostate cancer. Investig Clin Urol. 2019;60(1):4-13. doi:10.4111/icu.2019.60.1.4 30637355 PMC6318202

[22] Streicher J, Meyerson BL, Karivedu V, Sidana A. A review of optimal prostate biopsy: indications and techniques. Ther Adv Urol. 2019;11:1756287219870074. 31489033 10.1177/1756287219870074 PMC6713958

[23] Fowler H, Belot A, Ellis L, . Comorbidity prevalence among cancer patients: a population-based cohort study of four cancers. BMC Cancer. 2020;20(1):2. doi:10.1186/s12885-019-6472-9 31987032 PMC6986047

[24] Bradley CJ, Dahman B, Anscher M. Prostate cancer treatment and survival: evidence for men with prevalent comorbid conditions. Med Care. 2014;52(6):482-489. doi:10.1097/MLR.0000000000000113 24824535 PMC4129542

[25] Gretzer MB, Partin AW. PSA levels and the probability of PCa on biopsy. *European Urology Supplements*. 2002;1(6):21-27. doi:10.1016/S1569-9056(02)00053-2

[26] Xia J, Trock BJ, Gulati R, . Overdetection of recurrence after radical prostatectomy: estimates based on patient and tumor characteristics. Clin Cancer Res. 2014;20(20):5302-5310. doi:10.1158/1078-0432.CCR-13-3366 25320374 PMC4203422

[27] Egbers N, Schwenke C, Maxeiner A, Teichgräber U, Franiel T. MRI-guided core needle biopsy of the prostate: acceptance and side effects. Diagn Interv Radiol. 2015;21(3):215-221. doi:10.5152/dir.2014.14372 25858525 PMC4463259

[28] Hjälm-Eriksson M, Ullén A, Johansson H, Levitt S, Nilsson S, Kälkner KM. Comorbidity as a predictor of overall survival in prostate cancer patients treated with external beam radiotherapy combined with HDR brachytherapy boosts. Acta Oncol. 2017;56(1):21-26. doi:10.1080/0284186X.2016.1253864 27882806

[29] Chang E, Jones TA, Natarajan S, . Value of tracking biopsy in men undergoing active surveillance of prostate cancer. J Urol. 2018;199(1):98-105. doi:10.1016/j.juro.2017.07.038 28728993 PMC5760302

[30] Cooperberg MR, Ramakrishna NR, Duff SB, . Primary treatments for clinically localised prostate cancer: a comprehensive lifetime cost-utility analysis. BJU Int. 2013;111(3):437-450. doi:10.1111/j.1464-410X.2012.11597.x 23279038 PMC3587031

[31] Roth JA, Gulati R, Gore JL, Cooperberg MR, Etzioni R. Economic analysis of prostate-specific antigen screening and selective treatment strategies. JAMA Oncol. 2016;2(7):890-898. doi:10.1001/jamaoncol.2015.6275 27010943 PMC4945414

[32] Heijnsdijk EA, Wever EM, Auvinen A, . Quality-of-life effects of prostate-specific antigen screening. N Engl J Med. 2012;367(7):595-605. doi:10.1056/NEJMoa1201637 22894572 PMC4982868

[33] Sullivan PW, Lawrence WF, Ghushchyan V. A national catalog of preference-based scores for chronic conditions in the United States. Med Care. 2005;43(7):736-749. doi:10.1097/01.mlr.0000172050.67085.4f 15970790

[34] Lobo JM, Trifiletti DM, Sturz VN, . Cost-effectiveness of the Decipher genomic classifier to guide individualized decisions for early radiation therapy after prostatectomy for prostate cancer. Clin Genitourin Cancer. 2017;15(3):e299-e309. doi:10.1016/j.clgc.2016.08.012 28089723

[35] Barnett CL, Davenport MS, Montgomery JS, Wei JT, Montie JE, Denton BT. Cost-effectiveness of magnetic resonance imaging and targeted fusion biopsy for early detection of prostate cancer. BJU Int. 2018;122(1):50-58. doi:10.1111/bju.14151 29388388

[36] de Carvalho TM, Heijnsdijk EAM, de Koning HJ. Comparative effectiveness of prostate cancer screening between the ages of 55 and 69 years followed by active surveillance. Cancer. 2018;124(3):507-513. doi:10.1002/cncr.31141 29231973 PMC6680244

[37] Hayes JH, Ollendorf DA, Pearson SD, . Observation versus initial treatment for men with localized, low-risk prostate cancer: a cost-effectiveness analysis. Ann Intern Med. 2013;158(12):853-860. doi:10.7326/0003-4819-158-12-201306180-00002 23778902 PMC4487888

[38] Sanders GD, Neumann PJ, Basu A, . Recommendations for conduct, methodological practices, and reporting of cost-effectiveness analyses: second panel on cost-effectiveness in health and medicine. JAMA. 2016;316(10):1093-1103. doi:10.1001/jama.2016.12195 27623463

[39] Marseille E, Larson B, Kazi DS, Kahn JG, Rosen S. Thresholds for the cost-effectiveness of interventions: alternative approaches. *Bull World Health Organ*. 2015;93(2):118-124. doi:10.2471/BLT.14.138206 PMC4339959 25883405

[40] Norris JM, Carmona Echeverria LM, Bott SRJ, . What type of prostate cancer is systematically overlooked by multiparametric magnetic resonance imaging? an analysis from the PROMIS cohort. Eur Urol. 2020;78(2):163-170. doi:10.1016/j.eururo.2020.04.029 32370911 PMC7397509

[41] Brown LC, Ahmed HU, Faria R, . Multiparametric MRI to improve detection of prostate cancer compared with transrectal ultrasound-guided prostate biopsy alone: the PROMIS study. Health Technol Assess. 2018;22(39):1-176. doi:10.3310/hta22390 30040065 PMC6077599

[42] de Rooij M, Crienen S, Witjes JA, Barentsz JO, Rovers MM, Grutters JPC. Cost-effectiveness of magnetic resonance (MR) imaging and MR-guided targeted biopsy versus systematic transrectal ultrasound–guided biopsy in diagnosing prostate cancer: a modelling study from a health care perspective. Eur Urol. 2014;66(3):430-436. doi:10.1016/j.eururo.2013.12.012 24377803

[43] Stangelberger A, Waldert M, Djavan B. Prostate cancer in elderly men. Rev Urol. 2008;10(2):111-119. 18660852 PMC2483315

[44] Neumann PJ, Kamal-Bahl S. Should value frameworks take a “societal perspective”? *Health Affairs*. September 6, 2017. Accessed September 21, 2021. https://www.healthaffairs.org/do/10.1377/hblog20170906.061833/full

[45] Riffenburgh RH, Amling CL. Use of early PSA velocity to predict eventual abnormal PSA values in men at risk for prostate cancer. Prostate Cancer Prostatic Dis. 2003;6(1):39-44. doi:10.1038/sj.pcan.4500614 12664063

[46] Shakir NA, George AK, Siddiqui MM, . Identification of threshold prostate specific antigen levels to optimize the detection of clinically significant prostate cancer by magnetic resonance imaging/ultrasound fusion guided biopsy. J Urol. 2014;192(6):1642-1648. doi:10.1016/j.juro.2014.08.002 25117476 PMC4684948

[47] Tooker GM, Truong H, Pinto PA, Siddiqui MM. National survey of patterns employing targeted MRI/US guided prostate biopsy in the diagnosis and staging of PCa. *Curr Urol*. 2018;12(2):97-103. doi:10.1159/000489426 "https://pubmed.ncbi.nlm.nih.gov/31114467" 31114467 PMC6504798

