# Supplementary material for: Cost-Effectiveness of Annual Prostate MRI and Potential MRI-Guided Biopsy After Prostate-Specific Antigen Test Results
Source: JAMA Netw Open. 2023 Nov 29;6(11):e2344856. doi: 10.1001/jamanetworkopen.2023.44856 (PMC10687655; doi:10.1001/jamanetworkopen.2023.44856)
Supplement: Supplement 1. — eAppendix. Details on Material and Methods eReferences. eTable. Mortality Inputs by PSA Strata Used in the Decision-Analytic Model eFigure 1. Decision-Analytic Models eFigure 2. Tornado Diagrams for PSA 2.5-4.0 ng/mL Stratum [file jamanetwopen-e2344856-s001.pdf]

Supplementary Online Content

Yun H, Kim J, Gandhe A, et al. Cost-effectiveness of annual prostate MRI and potential MRI-guided biopsy after prostate-specific antigen test results. *JAMA Netw Open.* 2023;6(11):e2344856. doi:10.1001/jamanetworkopen.2023.44856

eAppendix. Details on Material and Methods
eReferences.
eTable. Mortality Inputs by PSA Strata Used in the Decision-Analytic Model
eFigure 1. Decision-Analytic Models
eFigure 2. Tornado Diagrams for PSA 2.5-4.0 ng/mL Stratum

This supplementary material has been provided by the authors to give readers additional information about their work.

© 2023 Yun H et al. *JAMA Network Open.*

eAppendix. Details on Material and Methods

Details on Model Overview

In our decision-analytic Markov model, it was presumed that the population would be classified in their PSA strata from PSA screening and was eligible when they entered the model to receive either standard biopsy or MRI and potential MRI-guided biopsy. PSA is a protein that is produced by prostate cells and testing the level of PSA in the blood is used for prostate cancer screening. In general, higher PSA levels indicate a higher chance of having prostate cancer, and traditionally, PSA levels of 4.0 ng/mL and lower were considered normal.(1) Men in out cohort were assumed to get tested for the PSA screening before they entered the model, at age 65, and were stratified by their PSA levels. In the MRI and potential MRI-guided biopsy cohort, a diagnostic MRI examination is performed and only when the lesion(s) is/are detected, MRI-targeted biopsy proceeds. Those who are tested positive from the MRI get MRI-guided biopsy and those who got false negative test results from the MRI-guided biopsy are moved to the 'Missed PCa' stage where they will spend the next cycle and then get the MRI again in the following cycle based on our annual testing design. Those who are tested negative are moved to the 'Missed PCa' group. 'Missed PCa' group will be further divided into subgroups depending on their PCa risk-grade as well as the presence of comorbidities so that we can address the risk-grade and health-condition specific mortality. True negatives will reenter into the initial status in the following cycle. Cohort entered into each biopsy strategy arm (MRI and MRI-guided biopsy vs. standard biopsy) do not cross-treated by the other strategy throughout the simulation.

The population was stratified by four different initial PSA score strata: less than 2.5ng/mL, 2.5-4.0 ng/mL, 4.1-10.0 ng/mL, and greater than 10.0ng/mL. We examined the 2.5-4.0 ng/mL category separately because PSA scores >2.5 ng/mL indicate a higher probability of being diagnosed with cancer, recognizing that current clinical practice accounts for other risk factors in biopsy decisions for this group.(1-3) Patients with comorbidities, such as diabetes, chronic obstructive pulmonary disease, congestive heart failure, or multiple comorbid conditions that can be exacerbated by PCa treatment, may

© 2023 Yun H et al. *JAMA Network Open.*

defer prostate cancer treatment out of concern that it would not improve length or quality of life,(4) and it is also assumed that patients with comorbidities that severely limit life expectancy would not be screened for prostate cancer, where ‘comorbidities’ are the conditions which would have a greater impact on length and quality of life than the PCa itself, thus justifying deferral of PCa treatment.

Individuals with high grade (grade groups 3-5) and intermediate grade PCa (grade group 2) without comorbidities are presumed to undergo treatment(5). Individuals with low grade PCa (grade group 1) without comorbidities move to the ‘PCa Diagnosed+Surveillance’ health state for the following cycle in which they will receive annual surveillance with PSA and MRI. Those who have comorbidities precluding them from any treatment for PCa move to the ‘PCa Diagnosed+Comorbidities’ health state for the following cycle where they will no longer get screening for PCa but will incur health care costs attributable to the management of PCa and related conditions (see Table 2).

Individuals who have been treated are placed into either the remission or recurrence health state and they start their following cycle in the ‘PCa Diagnosed+Treated’ stage without further tests and accrue relevant health care costs as summarized in Table 2. As we are examining the cost-effectiveness of an annual biopsy for prostate cancer, patients who stayed in ‘Missed low-, intermediate-, or high-grade prostate cancer’ will go through the surveillance in the following year.

Details on Primary Measures

In our evaluation on the cost-effectiveness using ICERs, all future costs and QALYs were discounted at a rate of 3% annually in accordance with published guidelines (6). One-way sensitivity analyses were performed on key test characteristics, i.e. sensitivity and specificity of MRI, and MRI-guided biopsy, for values ranging from 10% to perfect accuracy. Probabilistic sensitivity analysis used Gamma distributions for costs and Beta distributions for quality-of-life weights and probabilities were applied (7). This analysis allows for simultaneous variation of all parameters to determine the proportion

© 2023 Yun H et al. *JAMA Network Open.*

of simulations in which each strategy was cost-effective at a threshold of $100,000/QALY, thereby addressing uncertainty in input values.

Details on Data Sources

Probabilities used in the model that were derived from the literature include: the probability of having PCa by PSA levels (8), the probability of having comorbidities in PCa patients (9), the probability of PCa recurrence after treatment depending on PSA levels (10) the probability of upgrading from the previous PCa grade to a higher grade, and the sensitivity and specificity in detecting clinically significant PCa of each test; however, the specificity of MRI-guided biopsy was defined as 1 based on clinical judgement, meaning that prostate cancer diagnosed by biopsy is never a false positive (Table 1). Due to slight differences in the model cohort and the patient population of the data source used to estimate probability of PCa by PSA level and the probability of upgrading PCa grades, these values were secondarily reviewed by clinical authors for accuracy and face validity. Mortality rates for the health states were also derived from the literature or adjusted based on the hazard ratios of death from all-causes for different PSA levels (Supplemental eTable 1) (11-14).

Table 2 shows the costs used to populate the model. Cost inputs are from the Medicare perspective and in 2020 U.S. dollars for each unit of care a person may incur in the model. The cost components of diagnostic and surgical procedures (i.e., standard biopsy, MRI-guided biopsy, MRI, pathologic evaluations, and treatments including prostatectomy, radiation therapy and brachytherapy) were ascertained from Medicare reimbursement rates and from the literature. PCa treatment in this model is assumed to include the three most common options in equal proportions: prostatectomy, (external beam) radiation therapy, and brachytherapy. Patients who undergo these treatments were assumed to incur an annual ongoing management cost for remission. This cost was determined by taking the mean of the annual cost for remission and biochemical failure of $1,776 adjusted for inflation to 2020 dollars (15). Treating PCa in patients with comorbidities may result in a net harm and represent misallocation of

© 2023 Yun H et al. *JAMA Network Open.*

healthcare resources as these patients are more likely to die from another condition or underlying disease than from PCa (16). In a study by Trogdon et al., the authors adapted the Klabunde index, a validated comorbidity measurement algorithm, to show that people with diagnosed PCa and comorbidities who did not undergo any PCa treatment within 12 months of diagnosis incurred 3-year median total costs of $2,118 ($706 per year) in 2020 US dollars (17). We used this to estimate the annual ongoing costs for patients with clinically significant PCa who forgo receiving treatment due to comorbidities.

The recommended measure of effectiveness in economic evaluations is the quality-adjusted-life-year (QALY) (18). Estimated quality-of-life weights for each health state were taken from published literature (Table 2). We adjusted the disutility of standard biopsy from Barnett et al. (2008) to account for differences between pain scores for MRI-guided biopsy versus standard biopsy reported by Egbers et al. (2015). The annual quality-of-life value of active surveillance, 0.97 (19), was used as the QALY decrement for having low or intermediate grade PCa because individuals with intermediate-grade PCa may be appropriate candidates for active surveillance (20). Concerns about the risk of mortality from PCa that would affect their quality of life should be relatively low in all these individuals.

© 2023 Yun H et al. *JAMA Network Open.*

eReferences.

1. “Prostate-Specific Antigen (PSA) Test - NCI.” National Cancer Institute, 11 March 2022, https://www.cancer.gov/types/prostate/psa-fact-sheet. Accessed 10 November 2022.
2. Heijnsdijk EA, Denham D, de Koning HJ. The Cost-Effectiveness of PCa Detection with the Use of Prostate Health Index. *Value Health*. 2016;19(2):153-157. doi:10.1016/j.jval.2015.12.002
3. Prostate Cancer Detection: PSA Screening. ZEROCANCER. https://zerocancer.org/learn/about-prostate-cancer/detection-diagnosis/psa-test/. Accessed February 8, 2021.
4. Fowler, H., Belot, A., Ellis, L. et al. Comorbidity prevalence among cancer patients: a population-based cohort study of four cancers. *BMC Cancer* 20, 2 (2020). https://doi.org/10.1186/s12885-019-6472-9
5. Shah N, Ioffe V. Frequency of Gleason score 7 to 10 in 5100 elderly prostate cancer patients. *Rev Urol*. 2016;18(4):181-187. doi:10.3909/riu0732
6. Sanders GD, Neumann PJ, Basu A, et al. Recommendations for Conduct, Methodological Practices, and Reporting of Cost-effectiveness Analyses: Second Panel on Cost-Effectiveness in Health and Medicine. *JAMA*. 2016;316(10):1093–1103. doi:10.1001/jama.2016.12195
7. Briggs A. Probabilistic analysis of cost-effectiveness models: statistical representation of parameter uncertainty. *Value Health*. 2005;8(1):1-2. doi:10.1111/j.1524-4733.2005.08101.x
8. Gretzer MB, Partin AW. PSA Levels and the Probability of PCa on Biopsy. *European Urology Supplements*, vol. 1, no. 6, 1 Sept. 2002, pp. 21–27., doi:10.1016/s1569-9056(02)00053-2.
9. Hjälm-Eriksson M, Ullén A, Johansson H, Levitt S, Nilsson S, Kälkner KM. Comorbidity as a predictor of overall survival in prostate cancer patients treated with external beam radiotherapy combined with HDR brachytherapy boosts. *Acta Oncol*. 2017;56(1):21-26. doi:10.1080/0284186X.2016.1253864
10. Xia J, Trock BJ, Gulati R, et al. Overdetection of Recurrence after Radical Prostatectomy: Estimates Based on Patient and Tumor Characteristics. *Clinical Cancer Research*. 2014;20(20):5302-5310. doi:10.1158/1078-0432.ccr-13-3366
11. Arias E, Xu JQ. United States life tables, 2017. National Vital Statistics Reports; vol 68 no 7. Hyattsville, MD: National Center for Health Statistics. 2019.
12. Van Hemelrijck M, Folkvaljon Y, Adolfsson J, et al. Causes of death in men with localized PCa: a nationwide, population-based study. *BJU Int*. 2016;117(3):507-514. doi:10.1111/bju.13059
13. Sieh W, Lichtensztajn DY, Nelson DO, et al. Treatment and Mortality in Men with Localized PCa: A Population-Based Study in California. *Open Prost Cancer J*. 2013;6:1-9.
14. Tang P, Sun L, Uhlman MA, Polascik TJ, Freedland SJ, Moul JW. Baseline PSA as a predictor of prostate cancer-specific mortality over the past 2 decades: Duke University experience. *Cancer*. 2010;116(20):4711-4717. doi:10.1002/cncr.25447
15. Cooperberg MR, Ramakrishna NR, Duff SB, et al. Primary treatments for clinically localised prostate cancer: a comprehensive lifetime cost-utility analysis. *BJU Int*. 2013;111(3):437-450. doi:10.1111/j.1464-410X.2012.11597.x
16. VanderWeele DJ, Brown CD, Taxy JB, et al. Low-grade prostate cancer diverges early from high grade and metastatic disease. *Cancer Sci*. 2014;105(8):1079-1085. doi:10.1111/cas.12460
17. Trogdon JG, Falchook AD, Basak R, Carpenter WR, Chen RC. Total Medicare Costs Associated With Diagnosis and Treatment of Prostate Cancer in Elderly Men. *JAMA Oncol*. 2019;5(1):60-66. doi:10.1001/jamaoncol.2018.3701
18. Bravo VY, Sculpher M. Quality-adjusted life years. *Practical Neurology*. 2008;8:175-182.
19. Heijnsdijk EA, Wever EM, Auvinen A, et al. Quality-of-life effects of prostate-specific antigen screening. *N Engl J Med*. 2012;367(7):595-605. doi:10.1056/NEJMoa1201637.
20. Cooperberg MR, Cowan JE, Hilton JF, et al. Outcomes of active surveillance for men with intermediate-risk prostate cancer. *J Clin Oncol*. 2011;29(2):228-234. doi:10.1200/JCO.2010.31.4252

© 2023 Yun H et al. *JAMA Network Open.*

eTable. Mortality Inputs by PSA Strata Used in the Decision-Analytic Model

Parameter	Distribution	Value (Range)	References
Mortality of men aged 65 without diagnosis in PCa and any treatment in PSA < 2.5 stratum	Beta	0.016 (0.000, 0.130)	Tang et al. (2010) Supplemental Ref. 14
Mortality of men aged 65 without diagnosis in PCa and any treatment in PSA 2.5-4.0 stratum	Beta	0.013 (0.000, 0.150)	CMS NVSR (2019) Supplemental Ref. 11
Mortality of men aged 65 without diagnosis in PCa and any treatment in PSA 4.1-10.0 stratum	Beta	0.015 (0.000, 0.150)	Tang et al. (2010) Supplemental Ref. 14
Mortality of men aged 65 without diagnosis in PCa and any treatment in PSA >10.0 stratum	Beta	0.018 (0.000, 0.120)	Tang et al. (2010) Supplemental Ref. 14
Mortality of low or intermediate-grade PCa without treatment in PSA < 2.5 stratum	Beta	0.021 (0.000, 0.300)	Van Hemelrijck et al. (2016) Supplemental Ref.12
Mortality of low or intermediate-grade PCa without treatment in PSA 2.5-4.0 stratum	Beta	0.017 (0.000, 0.350)	Van Hemelrijck et al. (2016) Supplemental Ref. 12
Mortality of low or intermediate-grade PCa without treatment in PSA 4.1-10.0 stratum	Beta	0.019 (0.000, 0.280)	Van Hemelrijck et al. (2016) Supplemental Ref. 12
Mortality of low or intermediate-grade PCa without treatment in PSA >10.0 stratum	Beta	0.023 (0.000, 0.270)	Van Hemelrijck et al. (2016) Supplemental Ref. 12
Mortality of low-grade PCa with comorbidities in PSA <2.5 stratum	Beta	0.041 (0.013, 0.099)	Van Hemelrijck et al. (2016) Supplemental Ref. 12
Mortality of low-grade PCa with comorbidities in PSA 2.5-4.0 stratum	Beta	0.033 (0.009, 0.086)	Van Hemelrijck et al. (2016) Supplemental Ref. 12
Mortality of low-grade PCa with comorbidities in PSA 4.1-10.0 stratum	Beta	0.037 (0.010, 0.086)	Van Hemelrijck et al. (2016) Supplemental Ref. 12
Mortality of low-grade PCa with comorbidities in PSA > 10.0 stratum	Beta	0.045 (0.028, 0.104)	Van Hemelrijck et al. (2016) Supplemental Ref. 12
Mortality of high-grade PCa with comorbidities in PSA <2.5 stratum	Beta	0.054 (0.014, 0.133)	Van Hemelrijck et al. (2016) Supplemental Ref. 12
Mortality of high-grade PCa with comorbidities in PSA 2.5-4.0 stratum	Beta	0.043 (0.016, 0.090)	Van Hemelrijck et al. (2016) Supplemental Ref. 12
Mortality of high-grade PCa with comorbidities in PSA 4.1-10.0 stratum	Beta	0.048 (0.019, 0.094)	Van Hemelrijck et al. (2016) Supplemental Ref. 12
Mortality of high-grade PCa with comorbidities in PSA > 10.0 stratum	Beta	0.059 (0.017, 0.092)	Van Hemelrijck et al. (2016) Supplemental Ref. 12

© 2023 Yun H et al. *JAMA Network Open.*

Mortality of PCa patients who have comorbidities precluding from treatment in PSA < 2.5 stratum	Beta	0.047 (0.003, 0.151)	Van Hemelrijck et al. (2016) [Supplemental Ref. 12]
Mortality of PCa patients who have comorbidities precluding from treatment in PSA 2.5-4.0 stratum	Beta	0.038 (0.001, 0.154)	Van Hemelrijck et al. (2016) [Supplemental Ref. 12]
Mortality of PCa patients who have comorbidities precluding from treatment in PSA 4.1-10.0 stratum	Beta	0.043 (0.018, 0.150)	Van Hemelrijck et al. (2016) [Supplemental Ref. 12]
Mortality of PCa patients who have comorbidities precluding from treatment in PSA > 10.0 stratum	Beta	0.052 (0.004, 0.165)	Van Hemelrijck et al. (2016) [Supplemental Ref. 12]
Mortality of PCa after treatment in PSA < 2.5 stratum	Beta	0.033 (0.000, 0.257)	Sieh et al. (2013) Supplemental Ref. 13
Mortality of PCa after treatment in PSA 2.5-4.0 stratum	Beta	0.027 (0.000, 0.330)	Sieh et al. (2013) Supplemental Ref. 13
Mortality of PCa after treatment in PSA 4.1-10.0 stratum	Beta	0.030 (0.000, 0.280)	Sieh et al. (2013) Supplemental Ref. 13
Mortality of PCa after treatment in PSA > 10.0 stratum	Beta	0.037 (0.000, 0.290)	Sieh et al. (2013) Supplemental Ref. 13
Mortality of high-grade PCa without treatment in PSA < 2.5 stratum	Beta	0.038 (0.000, 0.434)	Van Hemelrijck et al. (2016) [Supplemental Ref. 12]
Mortality of high-grade PCa without treatment in PSA 2.5-4.0 stratum	Beta	0.030 (0.000, 0.490)	Van Hemelrijck et al. (2016) [Supplemental Ref. 12]
Mortality of high-grade PCa without treatment in PSA 4.1-10.0 stratum	Beta	0.034 (0.000, 0.420)	Van Hemelrijck et al. (2016) [Supplemental Ref. 12]
Mortality of high-grade PCa without treatment in PSA >10.0 stratum	Beta	0.042 (0.000, 0.460)	Van Hemelrijck et al. (2016) [Supplemental Ref. 12]

Note: PSA = prostate-specific antigen. PCa = Prostate Cancer. Mortality for different PSA strata other than PSA 2.5-4.0ng/mL stratum were calculated using Hazard Ratio of death from all causes for each PSA stratum from Tang et al. (2010), based on the probability of death under each condition in PSA 2.5-4.0 ng/mL. HRs for PSA 4.0-9.9ng/mL and PSA ≥ 10ng/mL have insignificant p-values (p-values = 0.502, 0.420, respectively). Mortalities derived from Sieh et al. (2013) and Van Hemelrijck et al. (2016) were calculated to annual probabilities. Values in the range column were collected from the Probabilistic Sensitivity Analyses results.

© 2023 Yun H et al. *JAMA Network Open.*

eFigure 1A. Decision-Analytic Model: Standard Biopsy Strategy

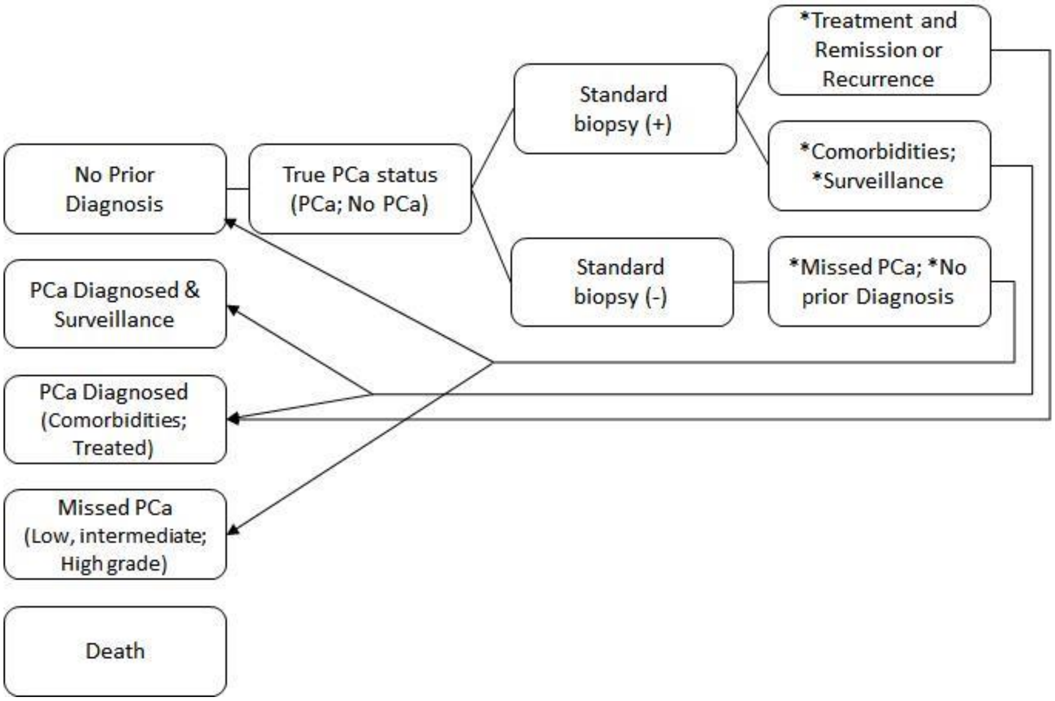

eFigure 1B. Decision-Analytic Model: MRI and Potential MRI-Guided Biopsy Strategy

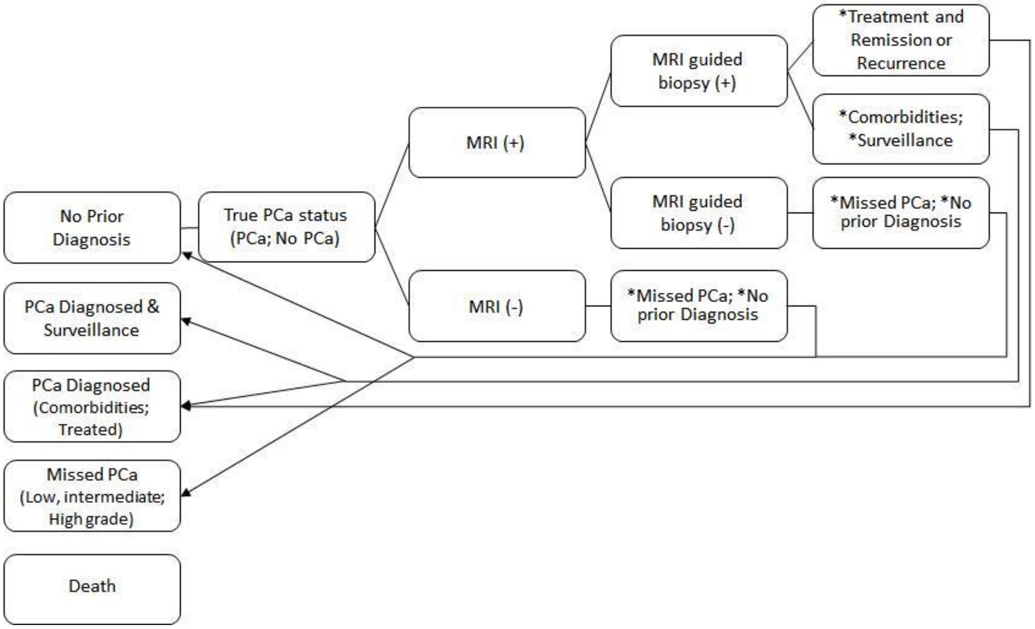

Note: Separate models were constructed for four levels of PSA result; less than 2.5 ng/mL, 2.5 to 4.0 ng/mL, 4.1 to 10.0 ng/mL, and greater than 10 ng/mL. * Asterisks: End states of one cycle and starting stages for the next cycle. Model details are available from the authors upon request.

© 2023 Yun H et al. *JAMA Network Open.*

eFigure 1C. Decision-Analytic Model: Key Structure of the Actual Decision-Analytic Model

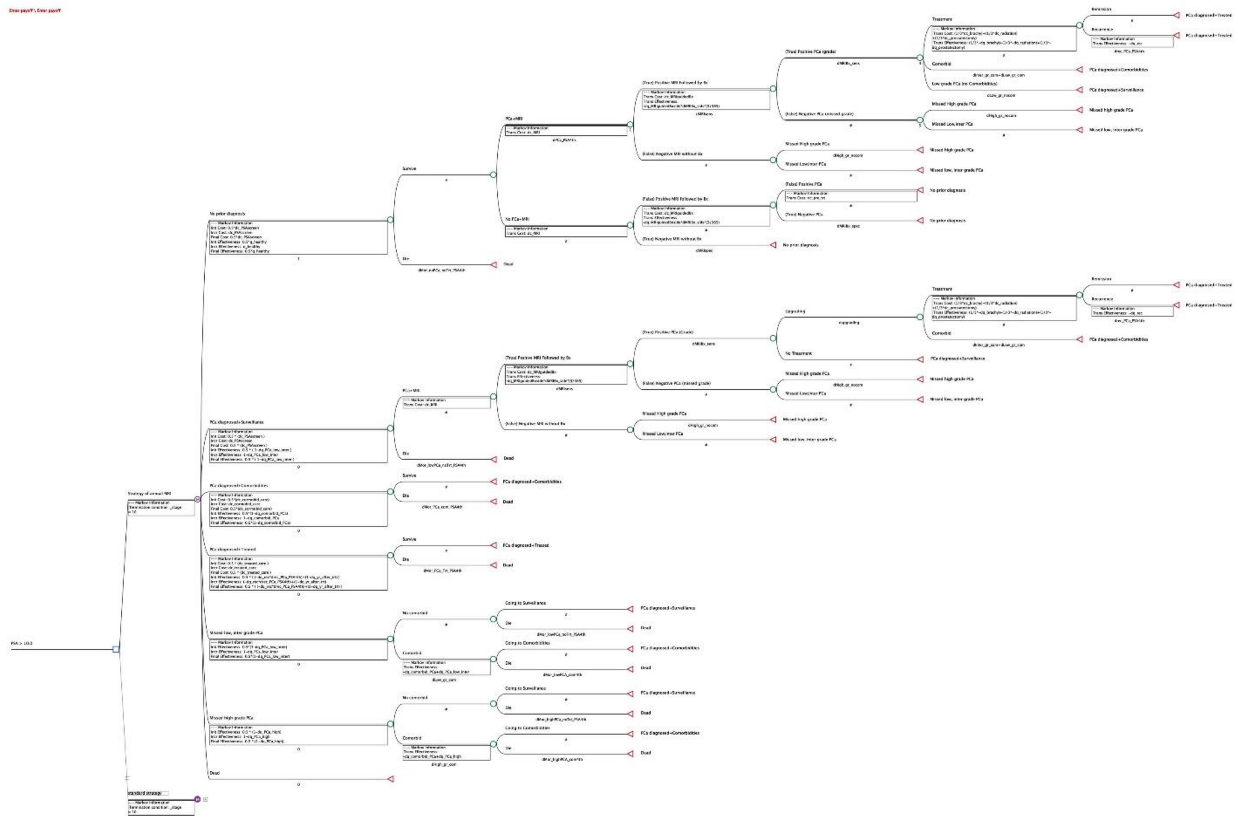

Figure 1C. Key structure of the actual model: A cycle of a decision tree for the cohort without prior diagnoses of PCa under the MRI and potential MRI-guided biopsy strategy was presented. Those who tested positive for the MRI get MRI-guided biopsy, and then those who tested positive for the MRI-guided biopsy either get treatment, move to surveillance for the next cycle without getting treatment, or move to Comorbidities management for the next cycle without getting treatment due to their low risk-grades of PCa or health conditions (comorbidities precluding them from getting treatment). Treated people move to Treatment management for the next cycle. People in comorbidities or post-treatment management status will remain in that status until they die without further surveillance. Those who got false-negative test results are moved to the Missed PCa group where they will spend the next cycle and then get the MRI in the following cycle based on our annual testing design. Missed PCa group will be further divided into subgroups depending on their PCa risk-grade as well as the presence of comorbidities so that we can address the risk-grade and health condition specific mortality. True negatives will reenter into the initial status in the following cycle. For the standard biopsy strategy, MRI and potential MRI-guided biopsy steps are replaced with a single standard biopsy.

© 2023 Yun H et al. *JAMA Network Open.*

eFigure 2A. Tornado Diagram for PSA 2.5 – 4.0 ng/mL Stratum: Incremental Effectiveness of Annual Prostate MRI and Possible MRI-Guided Biopsy vs Standard Biopsy

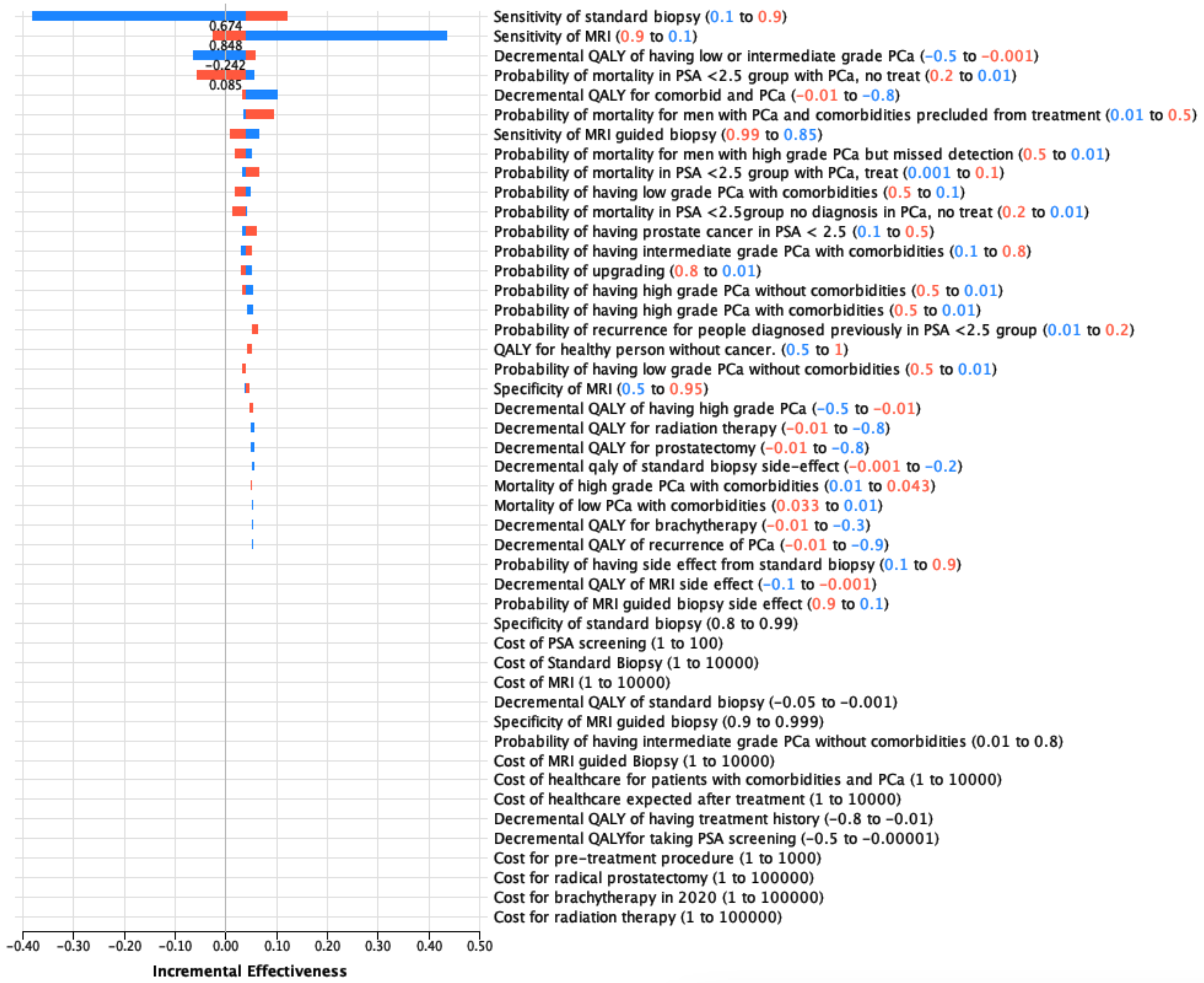

Note. The above tornado diagram illustrates the impact of directly varying input parameters within the probabilistic parameter sensitivity analysis (PSA) on the incremental effectiveness between annual prostate MRI and potentially followed by MRI-guided biopsy and standard biopsy for patients in the PSA 2.5 – 4.0 ng/mL stratum. The PSA 2.5 – 4.0 ng/mL stratum was chosen since it was the threshold stratum for the cost-effectiveness results. The right panel delineates the description of each variable, while their respective value ranges are denoted within parentheses. In the left panel, distinct colors (blue and red) represent the direction of change in cost associated with each variable, and accompanying this, specific threshold values are numerically inscribed below the corresponding horizontal bars.

© 2023 Yun H et al. *JAMA Network Open.*

eFigure 2B. Tornado Diagram for PSA 2.5 – 4.0 ng/mL Stratum: Incremental Cost of Annual Prostate MRI and Possible MRI-Guided Biopsy vs Standard Biopsy

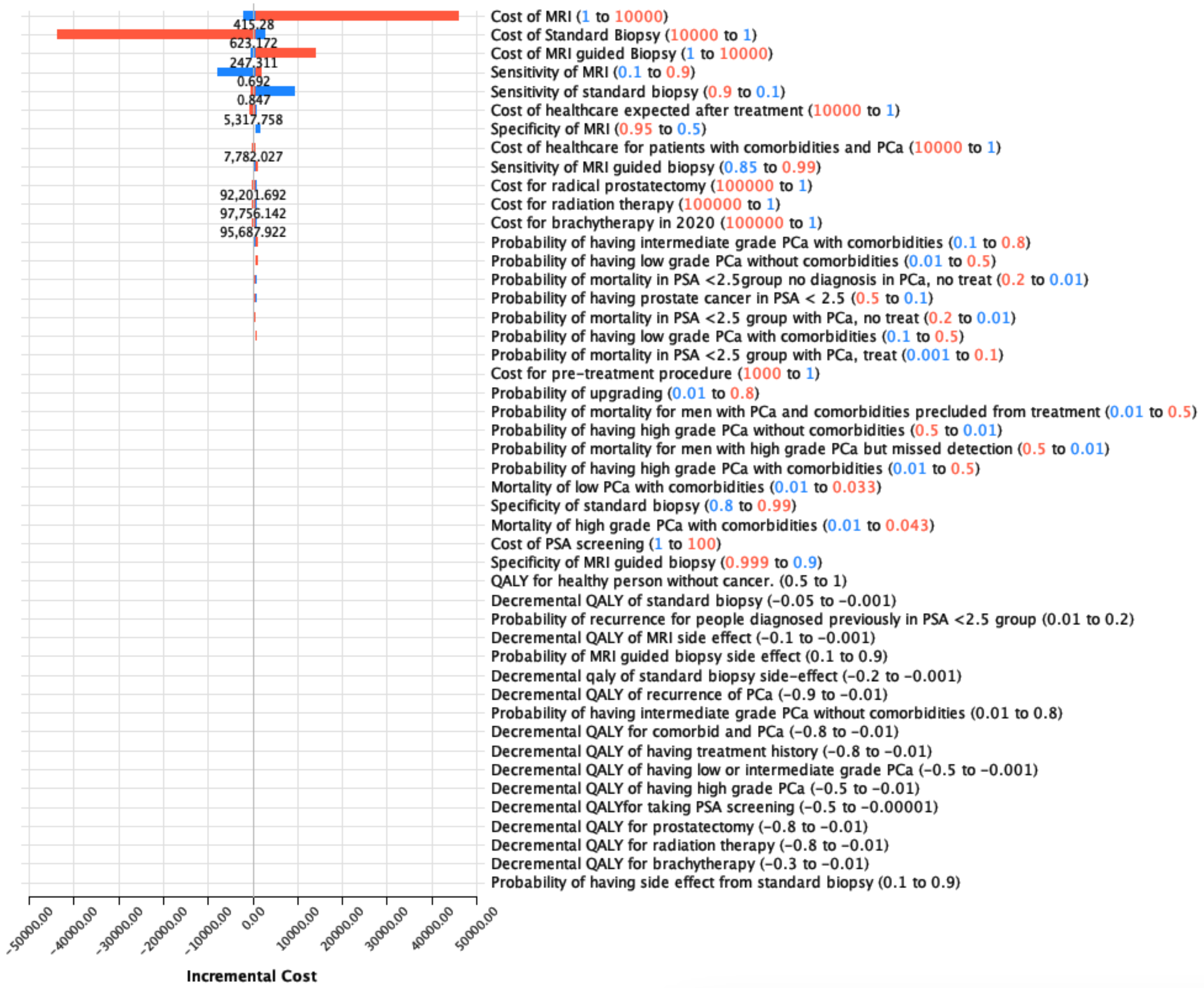

Note. The above tornado diagram illustrates the impact of directly varying input parameters within the probabilistic parameter sensitivity analysis (PSA) on the incremental cost between annual prostate MRI and potentially followed by MRI-guided biopsy and standard biopsy for patients in the PSA 2.5 – 4.0 ng/mL stratum. The PSA 2.5 – 4.0 ng/mL stratum was chosen since it was the threshold stratum for the cost-effectiveness results. The right panel delineates the description of each variable, while their respective value ranges are denoted within parentheses. In the left panel, distinct colors (blue and red) represent the direction of change in cost associated with each variable, and accompanying this, specific threshold values are numerically inscribed below the corresponding horizontal bars.

© 2023 Yun H et al. *JAMA Network Open.*
