# Supplementary material for: Cost-Effectiveness of Annual Prostate MRI and Potential MRI-Guided Biopsy After Prostate-Specific Antigen Test Results
Source: JAMA Netw Open. 2023 Nov 29;6(11):e2344856. doi: 10.1001/jamanetworkopen.2023.44856 (PMC10687655; doi:10.1001/jamanetworkopen.2023.44856)
Supplement: Supplement 2. — Data Sharing Statement [file jamanetwopen-e2344856-s002.pdf]

Data Sharing Statement

Yun. Cost-Effectiveness of Annual Prostate MRI and Possible MRI-Guided Biopsy After Prostate-Specific Antigen Test Results. *JAMA Netw Open*. Published November 29, 2023. doi:10.1001/jamanetworkopen.2023.44856

Data
Data available: Yes
Data types: Data (not involving human participants)
How to access data: All data used in the analysis will be published with the manuscript and in the supplemental appendix.
When available: With publication

Supporting Documents
Document types: None

Additional Information
Who can access the data: anyone requesting the data
Types of analyses: For any purpose.
Mechanisms of data availability: Data will be made available in the manuscript and online supplemental materials
Any additional restrictions: none.
